# Restinga ectomycorrhizae: a work in progress

**DOI:** 10.12688/f1000research.131558.1

**Published:** 2023-03-22

**Authors:** Ariadne N. M. Furtado, Marco Leonardi, Ornella Comandini, Maria Alice Neves, Andrea C. Rinaldi

**Affiliations:** 1 Departamento de Botânica, Campus Universitário Reitor João David Ferreira Lima, Universidade Federal de Santa Catarina, Florianópolis, Santa Catarina, 88040-960, Brazil; 2 Dipartimento di Scienze della Vita, della Salute e dell'Ambiente, Universita degli Studi dell'Aquila, L'Aquila, Abruzzo, I-67100, Italy; 3 Dipartimento di Scienze Biomediche, Universita degli Studi di Cagliari, Cagliari, Sardinia, I-09042, Italy

**Keywords:** Atlantic Forest, Brazilian fungi, diversity, ectomycorrhiza, fungal conservation, mycorrhizal symbiosis, Neotropics

## Abstract

**Background: **The Brazilian Atlantic Forest is one of the most biodiverse terrestrial ecoregions of the world. Among its constituents, restinga vegetation makes a particular case, acting as a buffer zone between the oceans and the forest. Covering some 80% of Brazilian coastline (over 7,300 km in length), restinga is a harsh environment where plants and fungi interact in complex ways that just now are beginning to be unveiled. Ectomycorrhizal symbiosis, in particular, plays a so far ungauged and likely underestimated role. We recently described the morpho-anatomical and molecular features of the ectomycorrhizae formed by several basidiomycetous mycobionts on the host plant *Guapira opposita*, but the mycorrhizal biology of restinga is still largely unexplored. Here, we report new data on the ectomycorrhizal fungal symbionts of *G. opposita*, based on the collection of sporomata and ectomycorrhizal root tips in restinga stands occurring in southern Brazil.

**Methods:** To obtain a broader view of restinga mycorrhizal and ecological potential, we compiled a comprehensive and up-to-date checklist of fungal species reported or supposed to establish ectomycorrhizae on restinga-inhabiting host plants, mainly on the basis of field observations.

**Results:** Our list comprises some 726 records, 74 of which correspond to putative ectomycorrhizal taxa specifically associated with restinga. These include several members of *Boletaceae*, *Amanita*, *Tomentella*/ *Thelephora*, *Russula*/ *Lactifluus*, and *Clavulina*, as well as hypogeous fungi, like the recently described *Longistriata flava*.

**Conclusions:** Our survey reveals a significant diversity of the restinga ectomycorrhizal mycobiota, indicating the importance of this symbiosis for the ecological functioning of a unique yet poorly known and threatened ecosystem.

## Introduction

Understanding how communities come together has been a primary goal of researchers over the last century. In addition to the diversity of organisms and their multiple strategies to resist environmental conditions, interspecific interactions add another layer of complexity to the structure of communities. ^1^ The mycorrhizal symbiosis is one of the most prominent and ecologically crucial mutualistic associations found in terrestrial habitats. ^2^ Plant and fungal partners interact in the rhizosphere, which contributes significantly to nutrient cycling and carbon sequestration. ^3^^,^^4^ The composition of mycorrhizal fungi in an ecosystem directly affects plant community structure, and environmental factors that influence species diversity over time have an impact on host selectivity for plant communities and fungal associations. ^5^

Research on arbuscular mycorrhizae (AM) is quite advanced in Brazil, with over 40 years of history. ^6^ However, our fascination with plant-fungus interactions took a different path allowing us to focus and investigate another intriguing mutualistic association, the ectomycorrhizae (ECM), from natural habitats of the Brazilian Atlantic Forest, particularly from the restinga. At the global scale, ectomycorrhizal plants have been documented in approximately 335 genera and 8,500 species, with recent research indicating that a large portion of plant symbionts have yet to be confirmed, specifically in the tropics. ^7^ On the other hand, ectomycorrhizal fungi have been assigned to 236 genera and approximately 20,000-25,000 species, ^8^ which is a small number when compared to current estimates of 2.2 to 3.8 million fungal species diversity. ^9^

In Brazil, ectomycorrhizal fungi often have a fragmented distribution due to the lack of information about them and because they do not always have the same distribution as the host plants. ^10^^,^^11^ This can result in high endemism at the species level due to the specific habitats they occupy. ^12^^–^^14^ In 2016, Roy and coworkers reported approximately 180 species of ectomycorrhizal fungi in Brazilian native forests. ^15^ In fact, the majority of the published studies were conducted in introduced *Pinus* and *Eucalyptus* plantations. ^14^^,^^16^^–^^18^ As a result, the diversity of ectomycorrhizal fungi associated with native plants of the Atlantic Forest still needs understanding. ^14^^,^^15^^,^^19^

The Atlantic Forest, which is home to several endemic species, is one of the world's top 25 priority areas for biodiversity conservation. ^20^ The high diversity of potentially ectomycorrhizal plant species suggests a large but still unknown diversity of ectomycorrhizal partners. ^11^ These relationships can be generalist, ^21^ however, there are cases of proven specificity, resulting from a long process of joint evolution between plants and fungi. ^5^ The Atlantic Forest includes ecosystems such as the restinga, where at least 700 specimens of typically ectomycorrhizal fungal taxa have already been collected. ^22^

The restinga was one of the first environments to be harmed by human intervention. Currently, 79% of the Brazilian coast is covered by restinga, representing an essential constituent of the Atlantic Forest. ^23^ However, in terms of biodiversity and conservation status, these ecosystems remain poorly understood. Restinga vegetation is associated with Quaternary coastal sand deposits and rocky coastal habitats, ^24^ grows on sandy soils near the sea between lake formations and/or dunes and, as it moves away from the ocean, is composed of creeping plants, shrubs and trees, including forming forests. ^25^

Due to the high frequency of symbiotic interaction in the restinga, ECM have a potentially critical role in restoration and management interventions in these ecosystems. ^26^ ECM can improve host plant resistance to pathogens through direct competition; ^27^ they can increase plant drought tolerance by improving plant-soil contact surface, host plant water conductivity, and resistance to high soil salinity by restricting sodium uptake by plant tissues and activating stress response pathways. ^28^ Sadly, anthropic activities are negatively affecting the diversity and functionality of the ectomycorrhizal community in forest soils due to soil erosion, changes in land use, inorganic toxins, fire, and non-native plant invasions. ^29^ Such processes have promoted the elimination of many populations and, potentially, the decrease of the genetic diversity of several species. ^30^ Nevertheless, these ecosystems are not considered priority conservation areas, and the high degree of degradation observed becomes especially harmful in a scenario of accelerated climate change. ^31^ Studies that aim to understand ectomycorrhizal interactions of restinga are important to help develop conservation and restauration projects. However, they are still in their early stages, especially because the majority of them are based solely on the presence of sporomata. To date, only two works have been published that link both fungus and plant partners in the restinga and present a detailed morphological and molecular characterization of the ECM ( *Hysterangium atlanticum* + *Coccoloba* spp. ^32^ and *Amanita viscidolutea* + *Guapira opposita* ^33^). Researches that have been developed in Brazil corroborate the urgent need to better understand the belowground diversity in the Atlantic Forest, especially considering that the restinga potentially harbors a unique community of mycorrhizal taxa. ^34^ In this work we present new information on ECM from restinga, through collections of basidiomata, as well as ectomycorrhizal root tips. Furthermore, based on survey data from national fungaria and herbaria, published literature, field observations, and molecular approaches, we provide a comprehensive and updated list of fungal species reported as (or supposed to) establishing ECM with native plants in the restinga. The data presented here reveal a high diversity of ectomycorrhizal fungi in the restinga and are discussed further below.

## Methods

### Restinga: definition and area extension

The restinga consists of a transition zone (ecotone) that acts as a buffer zone between the oceans and the forests and includes Holocene sands of marine origin. ^25^ Because of rapid leaching and the closeness of the ocean, the soil is nutrient-poor and water deficient, with high pH and it is highly salinized. ^35^ The community of ectomycorrhizal fungi, as well as plant symbionts, are structured and maintained in part by all of these factors. ^19^ Its vegetation is geologically young and originates from other ecosystems (Atlantic Forest, Amazon, Cerrado and Caatinga), but it exhibits phenotypic variation when compared to the habitat of origin, making it a unique, extreme ecosystem that requires specific adaptations and a high level of ecological plasticity. ^36^ As a result, the restinga diversity pattern varies greatly across its geographic range. ^37^

**Figure 1.**
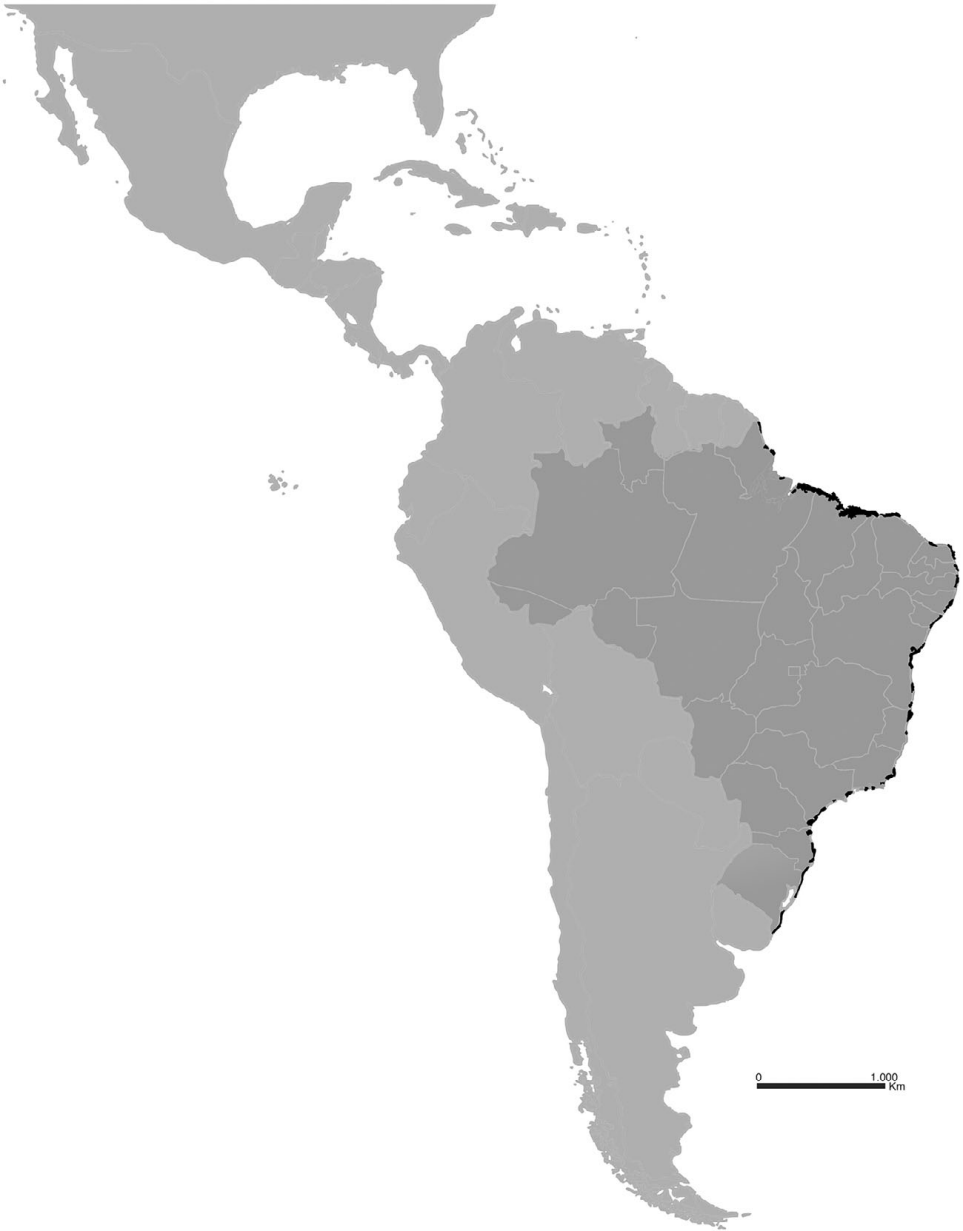
Map of restinga extension (in black) along the Brazilian Atlantic coast.

Restinga cover approximately 80% of the Brazilian coast, the equivalent to 7,360 Km in length, spanning all coastal states ( Figure 1). ^23^ In addition to providing habitat and refuge for many species for at least part of their life cycles, the restinga stores rainwater and assists in flood control and water cycle regulation. ^25^

### Collecting site and fungal sampling

Collections were made between October 2017 and May 2019 in three restinga areas in Florianópolis, Brazil: Parque Natural Municipal das Dunas da Lagoa da Conceição (-27.694028, -48.506587), Monumento Natural Municipal da Lagoa do Peri (-27.728243, -48.510175) and Parque Natural Municipal da Lagoa do Jacaré das Dunas do Santinho (-27.467783, -48.393395). The basidiomata were photographed in the field and identified by comparing them with the morphology described in the literature and by using DNA barcoding of the ITS region. Fungal species names were inspected in Index Fungorum (RRID:SCR_008975) and MycoBank (RRID:SCR_004950) for nomenclatural and taxonomic synonyms, and current names were adopted. After making morpho-anatomical analyses, the basidiomata were dried at 40 °C for further preservation. The identification of the plant was made by a botanist and confirmed by sequence similarity of the ITS region [ ^38^ Caddah personal communication].

For ECM, soil samples (approximately 20 cm ^3^) were collected below the basidiomata and left in water overnight. The roots were washed and carefully selected under a stereomicroscope. The roots that had an ectomycorrhizal mantle were morphotyped following the standard methodology and terminology used for studying ECM. ^39^ From each root system with a mantle, several tips were transferred to 70% alcohol and stored at -20 °C for subsequent DNA extraction. Also, part of the root system with the mantle was fixed in 4% glutaraldehyde for morpho-anatomical analyses. Voucher material of the basidiomata, ECM and the host plant are deposited in the FLOR herbarium and fungarium and the permanent collection of the mycology laboratory (Micolab) at the Universidade Federal de Santa Catarina (UFSC), Florianópolis, Brazil.

### Molecular identification of the fungi

Genomic DNA was extracted from the basidiomata using a PowerPlant®Pro DNA Isolation Kit (MO BIO Laboratories, Inc.), following the manufacturer's protocol adapted for fungi. The internal transcribed spacer of ribosomal nuclear DNA (nrITS) region was amplified using the primers ITS1F and ITS4 ^40^ and the following cycling parameters: an initial denaturation at 94 °C for 2 min; 40 cycles of 30 s at 94 °C, 45 s at 55 °C and 1 min at 72 °C; and a final extension at 72 °C for 7 min. A direct PCR approach was applied to amplify the ITS region from the ECM tips isolated from soil samples ^41^ using the same pair of primers (ITS1F/ITS4). A total of 2 ml of 20 mg/ml Bovine Serum Albumin (BSA) solution was added to each reaction tube to prevent PCR inhibition. The parameters applied to the PCR cycles followed Leonardi *et al.* ^42^ To identify the host plant, plant DNA was extracted from the ECM root tips using an isolation kit (see above), and the ITS region for the plant was amplified using the ITS-u1 and ITS-u4 primer pair ^38^ and the following cycle parameters: an initial denaturation at 94 °C for 4 min; 34 cycles of 30 s at 94 °C, 40 s at 55 °C and 1 min at 72 °C; and a final extension at 72 °C for 10 min. The DNA extracted from *Guapira opposita* leaves was used as a positive control. Sanger sequencing was performed with a BigDye Terminator 3.1 Cycle Sequencing Kit (Applied Biosystems, California, USA) at the company Myleus Biotecnologia, in Minas Gerais, Brazil, following the manufacturer’s instructions and using the same primers cited above for the correspondent symbionts. New sequences generated during this work were included in GenBank ^43^ and the accession numbers for the sequences are shown in Table 1.

**Table 1.** Ectomycorrhizal fungi reported to be associated with restinga ecosystems. Report of naturally occurring ectomycorrhizal fungi, potential host and/or sequence isolated from basidiomata or roots. All other records are about basidiomata collections, if not specified otherwise. Asterisk (*) refers to potential hosts which have not been confirmed. For names of fungal taxa and synonymy, we followed Index Fungorum ( http://www.indexfungorum.org/) and MycoBank ( http://www.mycobank.org).

Species	Host	Reference	Accession number
*Amanita coacta* Bas	*Psydium* sp. and *Ocotea* sp.*	^82^	-
	Guapira opposita*	This study	-
*Amanita crebresulcata* Bas	Euphorbiaceae, Fabaceae, Mimosaceae*	^80^	-
	-	^81^	-
	Guapira opposita*	This study	-
*Amanita lilloi* Singer	*Ficus* sp.*	^113^	-
*Amanita petalinovolva* Wartchow	Myrtaceae*	^88^	-
*Amanita psammolimbata* Wartchow & Sulzbacher	*Coccoloba* sp.*	^114^	-
*Amanita* sp. (FLOR61395)	-	^115^	KY769843
*Amanita* sp. (FLOR61397)	-	^115^	KY769853
*Amanita* sp. (FLOR61398)	-	^115^	KY769858
*Amanita viscidolutea* Menolli, Capelari & Baseia	*Coccoloba* sp.*	^85^	-
	*Guapira opposita*	^33^	MW000472; MW000473
	*Guapira opposita*	^33^ (ECM)	MW000471
	*Guapira opposita*	This study	-
	-	^14^	-
*Astraeus hygrometricus* (Pers.) Morgan	Chloroleucon foliolosum*	^116^	-
*Austroboletus festivus* (Singer) Wolfe	*Guapira opposita*	This study	-
	*Guapira opposita*	This study (ECM)	OP819290
	-	^110^	KY888002; KY888001; KY888000; KY887999; KY887998; KY886203; KY886202
*Boletellus cremeovelosus* A. Barbosa-Silva & Wartchow	*Coccoloba* spp.*	^77^	-
*Boletellus nordestinus* A.C. Magnago	Myrtaceae, *Coccoloba* spp.*	^75^	MG760443; MG760444; MG760442
*Boletinellus rompelii* (Pat. & Rick) Watling	-	^117^	-
	-	^118^	-
*Brasilioporus olivaceoflavidus* A.C. Magnago	Coccoloba, Guapira, Pisonia*	^74^	NG088318; OM068900; OM068913; OM068904; OM160556; OM160566; OM068912; OM068903; OM160555; OM160565; OM160576
*Brasilioporus simoniarum* A.C. Magnago	*Guapira* spp. *	^74^	OM068914; OM068905; OM160557; OM160567; OM160577; NG088319
*Cantharellus aurantioconspicuus* Wartchow & Buyck	-	^92^	-
*Cantharellus guyanensis* Mont.	Nyctaginaceae, Poligonaceae*	^91^	-
	-	^115^	KY769833
*Cantharellus protectus* Wartchow & F.G.B. Pinheiro	*Coccoloba* sp.*	^119^	-
*Clavulina amazonensis* Corner	-	^120^	-
*Clavulina incrustata* Wartchow	*Coccoloba* sp.*	^99^	-
*Clavulina incrustata* as *C. paraincrustata* Meiras-Ottoni & Gibertoni	-	^100^	KX811201; KX811196
*Clavulina junduensis* L.M. Ferst, A.N.M. Furtado & M.A. Neves	Guapira opposita*	^94^	MZ092867; MZ092866
	Guapira opposita*	This study	-
*Clavulina puigarii* (Speg.) Corner	-	^79^	-
*Coltricia focicola* (Berk & M.A. Curtis) Murrill	-	^121^	-
*Coltricia permollis* Baltazar & Gibertoni	-	^121^	-
	-	^122^	-
*Coltriciella oblectabilis* (Lloyd) Kotl., Pouzar & Ryvarden	-	^79^	-
*Craterellus niger* Sá, Pinheiro & Wartchow	-	^123^	-
*Entoloma aripoanum* Dennis	-	^124^	-
*Entoloma luteosplendidum* E. Horak & Cheype	-	^71^	-
*Entoloma tucuchense* Dennis	-	^124^	-
*Fistulinella ruschii* A.C. Magnago	Fabaceae*	^125^	KY886206; KY888006
	-	^126^	-
*Gloeocantharellus aculeatus* Linhares, P.P. Daniëls & M.A. Neves	-	^127^	KU884897; KU884889; KU884896; KU884888; KU884895; KU884887
*Gloeocantharellus substramineus* Wartchow	Myrtaceae, Rubiaceae, Poaceae, Euphorbiaceae*	^128^	-
*Gymnopus atlanticus* V. Coimbra & Wartchow	-	^73^	KT222654; KT222659
*Gymnopus montagnei* (Berk.) Redhead	-	^73^	KT222652; KT222653
*Gymnopus talisiae* V. Coimbra & Wartchow	-	^73^	KT222655; KT222656; KT222657; KT222658
*Hydnum villipes* Lloyd	-	^129^	-
*Hydnum pulcherrimum* Berk. & M.A. Curtis	-	^129^	-
*Hydropus griseolazulins* F.G.B. Pinheiro, Sá & Wartchow	Myrtaceae, Rubiaceae, Poaceae, Euphorbiaceae*	^72^	-
*Hysterangium atlanticum* Sulzbacher, Grebenc, Baseia et Nouhra	*Coccoloba alnifolia* and *Coccoloba laevis*	^32^	LT623206; LT623204; LT623205; LT635647; LT635648; LT635645; LT635646
	*Coccoloba alnifolia* and *Coccoloba laevis*	^32^ (ECM)	LT623210; LT623207; LT623208
*Inocybe* sp.	*Guapira opposita*	This study (ECM)	OP819291
*Lactifluus batistae* Wartchow, J.L. Bezerra & M. Cavalc.	Fabaceae subfam. Caesalpinoideae*	^130^	-
*Lactifluus dunensis* Sá & Wartchow	-	^131^	-
*Lactifluus neotropicus* (Singer) Nuytinck	-	^132^	MK937543; MK937563; MK937544; MK937564; KY769840;
*Lactifluus venosellus* Silva-Filho, Sá & Wartchow	Polygonaceae and Fabaceae*	^133^	MK929292
*Longistriata flava* Sulzbacher, Orihara, Grebenc, M.P. Martín & Baseia	*Coccoloba alnifolia, C. laevis* and Guapira*	^67^	LT574840; LT574842; LT574844; LT574839
*Nevesoporus nigrostipitatus* A.C. Magnago	*Coccoloba* and Guapira*	^74^	OM068918; OM068910; OM160562; OM068919; OM068911
*Phlebopus beniensis* (Singer & Digilo) Heinem. & Rammeloo	-	^134^	-
	-	^135^	-
*Phlebopus brasiliensis* Singer	Coccoloba laevis*	^136^	-
*Phlebopus portentosus* (Berk. & Broome) Boidjin	-	^118^	-
*Restingomyces reticulatus* Sulzbacher, B.T. Goto & Baseia	*Caesalpinia echinata, Lafoensia pacari* and Eugenia luschnathiana*	^137^	LT009410; LT009408; LT009409; LT009411; LT009412
*Russula pluvialis* Singer	Dicotiledoneas*	^48^^,^^138^	-
*Russula puiggarii* (Speg.) Singer	-	^115^	KY769834; KY769837
	Guapira opposita*	^34^	-
	Guapira opposita*	This study	-
*Sebacina aureomagnifica* Wartchow, Sulzbacher &Ovrebo	*Coccoloba alnifolia* and Coccoloba laevis*	^139^	LN868949; LN868950
*Thelephora palmata* (Scop.) Fr.	-	^79^	-
*Thelephora* sp.1	*Guapira opposita*	This study (ECM)	OP819292
*Tomentella* sp.1	*Guapira opposita*	This study (ECM)	OP819288
*Tomentella* sp.2	*Guapira opposita*	This study (ECM)	OP819289
*Tomentella* sp.3	*Guapira opposita*	This study (ECM)	OP819293
*Tomentella* sp.4	*Guapira opposita*	This study (ECM)	OP819286
*Tomentella* sp.5	*Guapira opposita*	This study (ECM)	OP819287
*Tomentella* sp. 6	*Guapira opposita*	This study (ECM)	OP819294
*Trechispora brasiliensis* (Corner) K.H. Larss.	-	^140^	-
*Trechispora copiosa* Meiras-Ottoni & Gibertoni	-	^141^	MN701013; MN687971
*Trechispora regularis* (Murrill) Liberta	-	^140^	MT406381; MH279999
*Trechispora thelephora* (Lév.) Ryvarden	Guapira opposita*	^34^ (ECM)	KY769825; KY769820
*Trechispora thelephora*	Guapira opposita*	^34^	KY769868
*Tylopilus aquarius var. megistus*	-	^77^	-
*Tylopilus dunensis* A.C. Magnago & M.A. Neves	-	^142^	MF113419; MF113428; MF113418; MF113420
*Tylopilus nigripes* A. Barbosa-Silva & Wartchow	*Coccoloba* sp.*	^143^	-
*Tylopilus* sp.	-	^142^	MF113424; MF113432; MF113425; MF113426; MF113427
*Xerocomus hypoxanthus* Singer	-	^144^	-
*Xerocomus* sp.	-	^144^	-

### Developing the record list

The data provided here on the relationship between restinga plants and ectomycorrhizal fungi are mainly based on field observations reports. Personal observations and information collected from a wide range of published and platform sources are included in the data source. The Brazilian fungaria collections (through SpeciesLink network) and available literature databases (e.g., Scopus (RRID:SCR 022559), PubMed (RRID:SCR 004846), ISI Web of Science (RRID:SCR 022706), ResearchGate (RRID:SCR 006505)) were searched for records on potential restinga host plants and related mycobionts. Only species belonging to fungal genera for which the ectomycorrhizal status has been proved or is considered likely were considered for listing. ^8^^,^^11^^,^^44^ Listed sequences of restinga ECM fungi ( Table 1) are those reported in relevant publications and were retrieved from either GenBank or UNITE. Some of the punctual ectomycorrhizal records were based only on the presence of the sporome next to a known plant symbiont, without any direct confirmation of the presence of ectomycorrhiza. As a result, these data are susceptible to non-measurable errors, particularly when more than one potentially ectomycorrhizal plant is in the environment. Despite our efforts to scan as many bibliographic sources as possible, our survey may be partial and incomplete.

## Results and Discussion

### Ectomycorrhizae in the Neotropics: a different story

Rolf Singer, a true pioneer in the study of mycorrhiza biology in South America, once wrote that he believed three ‘ectotrophic regions’ did exist in the continent, each one characterized by a single host plant genus. In a seminal work for the field, he listed the *Quercus humboldtii* area in Colombia, the ecosystem formed by *Alnus jorullensis* in the Andes and, more extensively, the *Nothofagus* region in Chile and Argentina. ^45^ Beyond these areas, Singer stated, ectomycorrhizal symbiosis in South America was restricted to plantations of imported trees, in particular *Pinus.* ^46^ However, as his knowledge of various types of forests in temperate and tropical South America improved, Singer expanded his view. “Our own recent investigation in the Lower Rio Negro region of Central Amazonia show, that certain vegetation types (campina, campinarana, igapó) are rich in ectomycorrhiza-forming fungi, *e.g.*, Boletaceae … Thus, in both hemispheres, certain tropical soils require for the formation of any kind of forest the presence of ectomycorrhiza,” Singer remarked in 1979. ^47^^,^^48^

Since Singer’s times, our understanding of the distribution, relevance and role of ectomycorrhizal symbiosis in many ecological settings in temperate, tropical and subtropical South America has grown considerably, but not so rapidly as one could have expected given the premises. Indeed, while Singer and colleagues just supposed the ectomycorrhizal status of many fungal species and relevant host plants on the basis of field observations, detailed studies able to identify and describe fungal structures on the roots of ectotrophic plants in most South American ecosystems began only in the last decade of the twentieth century, *i.e.*, considerably later than in the Northern Hemisphere. ^49^ According to the recent account of the currently known biogeographic pattern of ectomycorrhizal symbiosis in South America by Nouhra and colleagues, ^49^ three main regions can be recognized, broadly confirming Singer’s vision but also elaborating on it: 1) the Northern Andean cordillera, with mostly temperate forests, where ECM such as *Quercus*, *Colombobalanus*, *Alnus* and *Salix* occur; 2) the sub Antarctic forests in far Southern America, dominated by ectomycorrhizal trees in the *Nothofagaceae* ( *Fuscospora*, *Lophozonia* and *Nothofagus*); the Guiana Shield region and the coastal vegetation of the Atlantic rainforests of Brazil, where a large (and fast growing) number of ectomycorrhizal fungi in the/cortinarius, /russula-lactarius, /amanita and/clavulina lineages have been spotted in recent times with their associated host plants, including *Dicymbe*, *Aldina* ( *Fabaceae*), *Pseudomonotes* ( *Dipterocarpaceae*), *Pakaraimaea* ( *Cistaceae*), *Coccoloba* ( *Polygonaceae*), *Gnetum* ( *Gnetaceae*), *Pisonia*, *Neea*, and *Guapira* ( *Nyctaginaceae*). And is here that our story becomes more specific and very personal, as outlined below.

### Restinga mycorrhizae: more than it meets the eye

**Figure 2.**
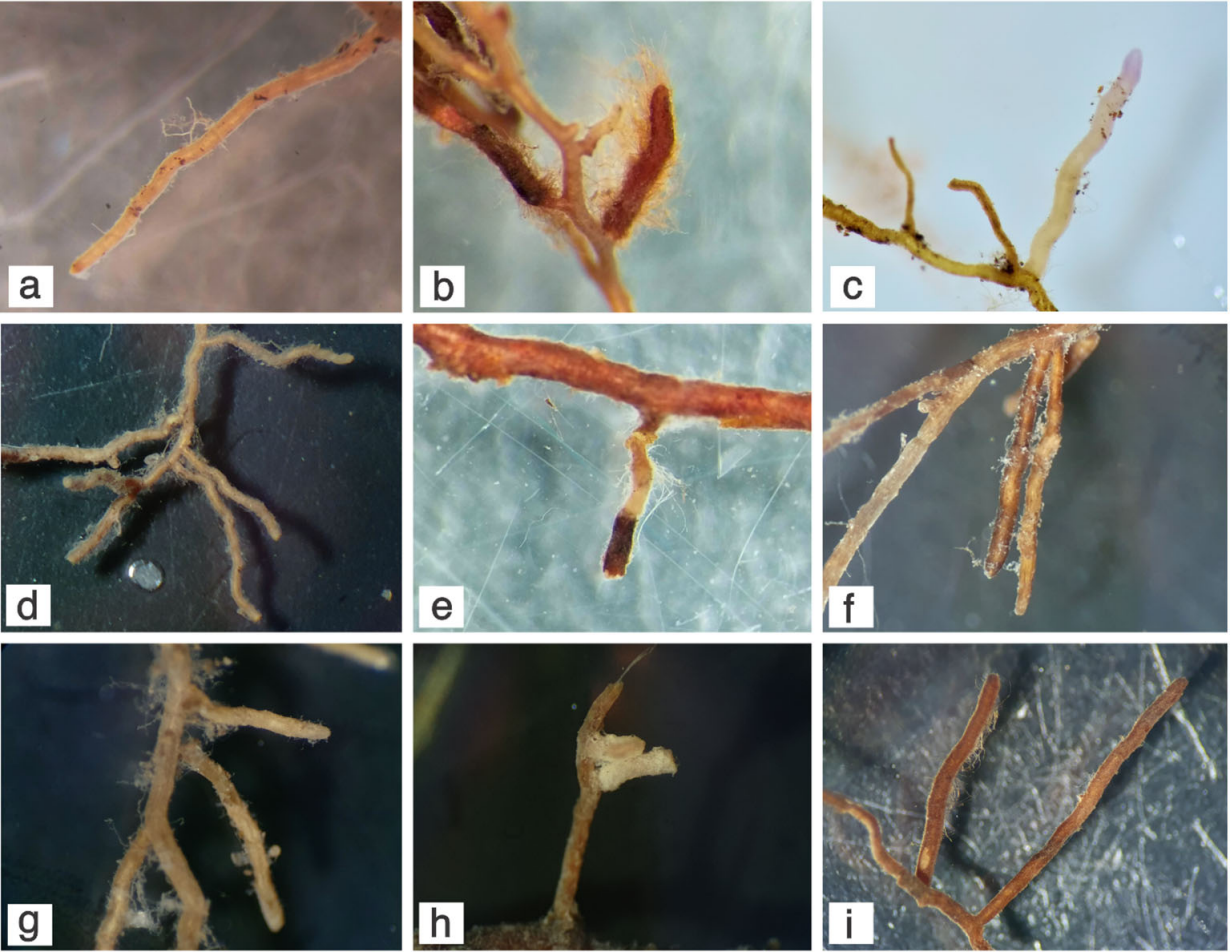
Ectomycorrhizal morphotypes associated with *Guapira opposita* roots in the restinga from South Brazil. (a) *Amanita viscidolutea*; (b) *Austroboletus festivus*; (c) *Inocybe* sp.; (d) *Thelephora* sp1.; (e) *Tomentella* sp1. and *Tomentella* sp2.; (f) *Tomentella* sp3.; (g) *Tomentella* sp4.; (h) *Tomentella* sp5.; (i) *Tomentella* sp6.

Mycorrhizal symbiosis plays a crucial role in basically each and every terrestrial ecosystem, ^3^ and restinga are not an exception. For many years, however, this peculiar coastal habitat has been the object of studies delving exclusively into the communities of arbuscular mycorrhizal fungi and their plant relationships, while almost no attention whatsoever has been devoted to the ectomycorrhizal component. We thus started investigating the spread, diversity and ecology of the ECM-fungal contingent, not only by recording the occurrence of sporomata of supposedly ectomycorrhizal macrofungi, but also looking directly at the roots and the structures therein. What we found was surprising, indeed. Working mainly at a restinga in the Parque Natural Municipal das Dunas da Lagoa da Conceição in Florianópolis, Brazil, we rapidly understood that *Guapira opposita* (Vell.) Reitz. is a hub for local ectomycorrhizal community, hosting a range of fungal species on its roots. Out of a total of 29 morphotypes collected from soil samples, 10 were found associated with *G. opposita* roots, all corresponding to Basidiomycota taxa, based on molecular barcoding. ^50^ The best represented clade was/tomentella-thelephora, with *Tomentella* bursting six species ( Table 1); of note, two macrofungi native species from the restinga of the Atlantic Forest, namely *Amanita viscidolutea* and *Austroboletus festivus*, besides occurring as basidiomata were also found associated to *G. opposita* roots in our survey ( Table 1). ^50^ The most striking characteristics of *Guapira* ECMs, however, remain with their morpho-anatomical features, that make them rather unique. The short, simple or long, thin branched ectomycorrhizal systems, close connections between the layered mantle and the cortical cells, absence of a Hartig net or other fungal elements in the cortex are diagnostic characteristics that make the *Guapira* ECMs we observed rather unique, to the point that we proposed the term ‘Guapirioid’ to distinguish them from the other known ectomycorrhizal types ( Figure 2). ^33^^,^^50^ Our study on the ectomycorrhiza of *A. viscidolutea* on *G. opposita* has been the first detailed morpho-anatomical and molecular characterization of a naturally occurring mycorrhiza associated with a native plant host in restinga forest in South America. ^33^ Besides *G. opposita*, we can also find members of the following potential ectomycorrhizal families growing in restinga: *Fabaceae*, *Moraceae*, *Myrtaceae*, *Nyctaginaceae*, *Polygonaceae*, and *Salicaceae.* ^51^

As mentioned above, representatives of/tomentella-thelephora clade were the most frequently encountered taxa in our restinga surveys, a finding in line with studies based on root and soil analysis that revealed that the/tomentella–thelephora clade is diverse and dominant in neotropical habitats, ^52^^,^^53^ although they are generally undersampled due to their inconspicuous basidiomata, which makes it difficult to identify the sampled taxon. It is known that members of *Nyctaginaceae* establish ectomycorrhizal associations with a low ECM fungal diversity in the Neotropics. ^19^^,^^54^ The mycorrhizal status of the family seems to be not homogeneous, with several species confirmed as dual-mycorrhizal ^55^^,^^56^ and many others believed to be non-mycorrhizal. ^7^^,^^57^ Moreover, some of the mycorrhizal structures observed in *Nyctaginaceae* escape classical classification. In the so-called ‘Pisonioid’ mycorrhizae of *Pisonia*, Hartig net is poorly or not developed, and instead ‘transfer cells’ are observable in the epidermis and cortex of the host root. ^58^^,^^59^ Haug *et al.*, ^60^ and Álvarez-Manjarrez *et al.*, ^61^ observed *Tomentella/Thelephora* and *Membranomyces* ectomycorrhizae associated with *Guapira* roots in Ecuador and Mexico, respectively. In both cases, they pointed to the presence of intraradical hyphae in the roots and the possibility of *Guapira* species forming a type of ectendomycorrhiza. On the basis of their work on the ectomycorrhizal types of *Nyctaginaceae* genera *Neea* and *Pisonia* in South Ecuador, Haug and colleagues ^54^ concluded that the set of observed characters (the combination of long root systems that are only partly transformed into ectomycorrhizae, with root hair formation that is not suppressed, occasional intracellular penetration of hyphae, and sporadic formation of transfer cell-like structures) may suggest that *Nyctaginaceae* represent an early step in the evolutionary change from arbuscular mycorrhization to ectomycorrhization. The ectomycorrhizal morphotypes we observed on *G. opposita* do not present the intraradical hyphae arrangement observed in other *Nyctaginaceae.* However, the absence of Hartig net recorded in our study is another indication of the plasticity and peculiarity of mycorrhizal biology of this host plant family. Of note, besides in *Pisonia*, the absence of Hartig net has been reported in the case of *Tremelloscypha* sp. and *Sebacina* sp. ectomycorrhizae on the roots of *Achatocarpus gracilis* Walter ( *Achatocarpaceae*, *Caryophyllales*) in a neotropical dry forest in Mexico. ^61^ Overall, this evidence points out the significance of *Caryophyllales* (that include the *Nyctaginaceae*) as mycorrhizal hosts in a variety of neotropical ecosystems, and the necessity to study the peculiar ectomycorrhizal associations and the role of ECM symbiosis in the Neotropics more thoroughly. ^62^ As for *G. opposita*, further work is currently underway in our lab, through *in vitro* synthesis of ECMs with selected mycobionts, to ascertain whether the formation of Guapirioid ECMs depends on the plant host, the fungal partner, or both.

“In this ecosystem where plants need to constantly deal with various environmental stresses, the symbiotic association of plants with arbuscular mycorrhizal fungi (AMF) is one of the main strategies for their survival, due to the ability of external fungal hyphae to absorb the scarce nutrients and water from the substrate, as well as hyphae contributing to soil aggregation … .and salinity tolerance,” noted da Silva and co-workers discussing the important ecological role played by AMF in restinga. ^63^ A host of studies conducted in the restinga across Brazil since the 1990s have indeed revealed many details of the AMF communities in these ecosystems, revealing that most of coastal dune plants investigated were associated with arbuscular mycorrhiza and that restinga AMF are significantly diverse. ^64^^,^^65^ In this context, it is relevant to note that *Guapira—*and likely other restinga host plants beyond *—*is a dual-mycorrhizal species, capable of hosting both arbuscular mycorrhizal and ectomycorrhizal associations. ^66^ Several ectomycorrhizal hosts share this feature, including *Eucalyptus*, *Alnus*, *Populus*, *Salix* and members of the *Cistaceae.* ^56^ They are typically plants that can survive in environments that are subject to severe disruptions like natural fire or even human activity, as well as soils deficient in nutrients. The benefits of dual-mycorrhizal colonization thus stretch from plants with increased rates of survival, growth, and nutrient absorption to environments, promoting establishment and increasing survival on unfavorable locations of linked AM/ECM plants. All these considerations fit potentially well with restinga characteristics.

### Restinga ectomycorrhizal fungi: connecting the dots

Our attempts to assess the diversity of ectomycorrhizal fungi associated with restinga, both through direct field sampling and by surveying records in the literature and in national fungaria, revealed 726 entries (Table 1S, which can be found as *Underlying data* ^23^). A total of 74 of these correspond to putative ectomycorrhizal taxa specifically associated with restinga, mostly derived from recent dedicated research and our own data ( Table 1). A total of 14 different taxa were recorded in our fieldwork in restinga fragments in southern Brazil; all are reported for the first time as linked to *Guapira opposita.* Several important ectomycorrhizal fungal taxa are represented in the list, with *Boletaceae* (15 spp.), *Amanita* (9 spp.), *Tomentella*/ *Thelephora* (8 spp.), *Russula*/ *Lactifluus* (7 spp.), and *Clavulina* (4 spp.). Three taxa of hypogeous fungi were recorded in the restinga, including the recently described *Longistriata flava* Sulzbacher, Orihara, Grebenc, M.P. Martín & Baseia, possibly associated with *Coccoloba* and *Guapira* spp. ^67^

Throughout our investigation, we isolated 10 distinct morphotypes from restinga fragments using random soil sampling ( Table 1). Basidiomata of equivalent species were also collected in two cases ( *Amanita viscidolutea* and *Austroboletus festivus*). Another eight species were collected only in association with host roots ( *Inocybe* sp., *Thelephora* sp. and six unknown species of *Tomentella*). The high frequency of *Thelephoraceae* representatives as fungal partners is remarkable in our data, as well as in other studies. ^52^^,^^68^ It is widely recognized that many species in this family are saprotrophs, however, it is possible that ectomycorrhizal species also occupy niches as saprotrophs to survive periods when they are not associated with the plant symbiont. ^69^ Previous studies indicate that many thelephoroid fungi associated with members of the Pisonieae tribe ( *Guapira*, *Neea* and *Pisonia*, except *P. grandis*) are generalists, as all telephoroid fungi found associated with members of Pisonieae were also found associated with other plant symbionts. ^68^^,^^70^ Ectomycorrhizal plants of the *Polygonaceae*, *Caesalpiniaceae* and *Fabaceae* families often occur in the same regions as the Pisonieae species, such that symbiosis in these species should also be examined. ^68^^–^^70^

Species of *Entoloma*, *Gymnopus*, *Hydropus*, and *Phlebopus* have been mentioned as ectomycorrhizal, ^12^ possibly associated with *Myrtaceae*, *Leguminosae*, *Rubiaceae*, *Polygonaceae*, and *Euphorbiaceae* in the restinga. ^71^^–^^74^ These putative ectomycorrhizal lineages, however, are not concentrated in specific clades neither form monophyletic groups of ectomycorrhizal isolates, ^12^ in such a way that their true ectomycorrhizal status must be confirmed. The genus *Boletus* is not recorded from Brazil (except from exotic plantations) but several *Tylopilus*, *Xerocomus* and *Phlebopus* species were originally deposited under the name *Boletus* sp. Taking this into account, we considered for listing records of *Boletus* sp. only from natural habitats.

### Restinga mycohighlights

**Figure 3.**
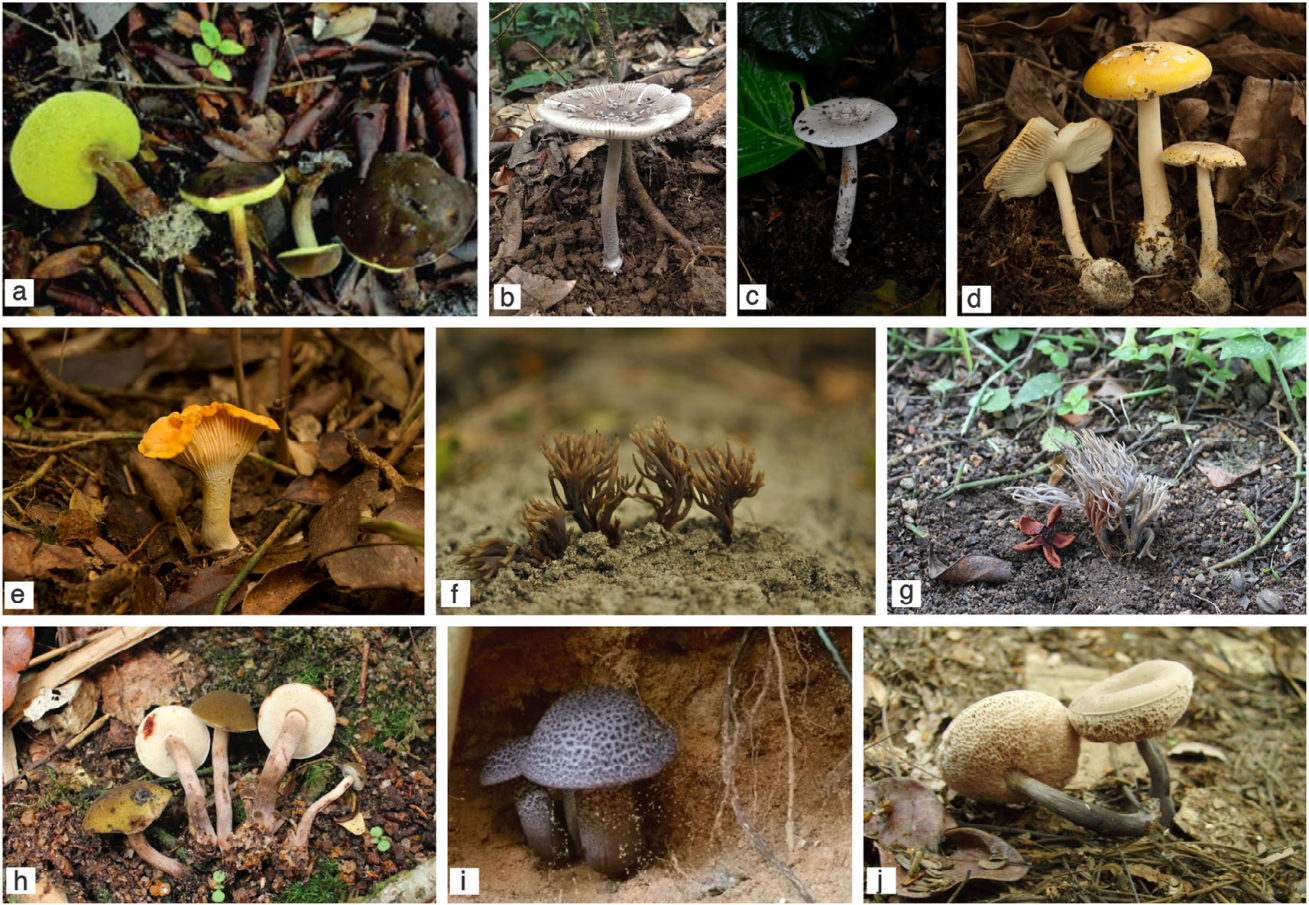
Basidiomata of selected species discussed in the text. (a) *Boletellus nordestinus*; (b) *Amanita crebresulcata*; (c) *Amanita coacta*; (d) *Amanita viscidolutea*; (e) *Cantharellus guyanensis*; (f) *Clavulina junduensis*; (g) *Clavulina incrustata*; (h) *Brasilioporus olivaceoflavidus*; (i) *Brasilioporus simoniarum*; (j) *Nevesoporus nigrostipitatus.* Photo credits (a) Eduardo Fazolino; (h; j) Altielys C. Magnago; (i) Juli Simon.

During our survey, we unearthed notable records of ectomycorrhiza-forming fungi occurring in the restinga that deserve special mention and additional notes ( Figure 3). As shown in Table 1, several boletoid taxa have been described as being associated with restinga, such as *Boletellus nordestinus* (MycoBank MB823951) ( Figure 3a). This species has been recently described from material collected in sandy soils in the northeast of Brazil, in the states of Paraíba and Rio Grande do Norte. ^75^ Although only found in two locations, it is expected that this species occurs in other restinga fragments along the Brazilian Atlantic coast. However, extensive searches in southern Brazil have been conducted, and the lack of records in these areas may indicate that this is a rare species [Altielys Magnago, personal communication]. *Boletellus nordestinus* can be distinguished from its closely related *Boletellus chrysenteroides* (Snell) Snell by its dry, velutinous, chocolate brown pileus, smaller basidiospores longitudinally ridged, dichotomously forked. ^75^ Also *B. chrysenteroides* is a North-American species that associates with oaks and hemlocks and grows in an unusual environment for boletus, in the midst of decayed wood. ^76^ Although the ECM hosts of *B. nordestinus* are unknown, specimens have been observed growing near confirmed ectomycorrhizal host plants: *Coccoloba alnifolia* Casar., *C. laevis* Casar. (Polygonaceae) and *Myrtaceae* species. ^5^ Currently, only four species of the genus are known from Brazil: *Boletellus ananas* var. *minor* Singer, *B. annas* var. *crassotunicatus* Singer have been described for the Amazon ^48^; *B. cremeovelosus* Barbosa-Silva & Wartchow ^77^ and *B. nordestinus* have been described for the Atlantic Forest. ^75^

*Amanita crebresulcata* (MycoBank MB308549) ( Figure 3b), *Amanita coacta* (MycoBank MB308546) ( Figure 3c) and *Amanita viscidolutea* (MycoBank MB514222) ( Figure 3d) were also noteworthy findings from our survey. The first two are *Vaginatae* sect. members with a patchy distribution, with *A. crebresulcata* found in the states of Amazonas, Mato Grosso (in the Brazilian Amazon), Paraíba, Paraná, Pernambuco and Santa Catarina (in the coastal Atlantic Forest), ^78^^–^^82^ and *A. coacta* found in Amazonas, São Paulo and Santa Catarina [ ^78^^,^^82^^,^^83^ as *A. crebresulcata*]. These records, however, may not reflect the true distribution of taxa in the country, as several regions remain under-sampled, and species records are based on specialists' areas of expertise. ^82^ Despite their macromorphological similarity, *A. coacta* is mainly characterized by having a felted submembranous volva, and the presence of a transverse belt-like portion of the volva that detaches from the saccade portion attached to the base of the stipe as it increases in length. ^84^ *Amanita crebresulcata*, on the other hand, has a thin saccade volva that usually breaks at the apex and leaves no remnants on top of the stipe. ^78^ *Amanita viscidolutea* was one of the most frequent fungi found during our field trips. Basidiomata of the species are vibrant yellow with slightly striated white pileus margin and the exannulate stipe. ^85^ The taxon belongs to the well-supported sect. *Amanita,* ^86^ reported to group important ectomycorrhizal taxa such as *Amanita muscaria* (L.) Lam. and *Amanita pantherina* (DC.) Krombh. ^87^ The species is known from restinga areas in the coastal Atlantic Forest from Rio Grande do Norte, where it was originally described, ^84^ Paraíba ^88^ and Santa Catarina. ^33^ Although it is usually found in relatively large populations, *A. viscidolutea* is a rare species and it grows in a specific type of vegetation that has been threatened by habitat loss and fragmentation by human population growth and expansion, along the Brazilian coast. ^25^ Using transmission electron microscopy, we recently described the morpho-anatomical characteristics of the ectomycorrhiza formed between *A. viscidolutea* and *Guapira opposita* roots; both partners were identified from the ectomycorrhizal root tips through molecular analyzes. ^33^

*Cantharellus guyanensis* (MycoBank MB240517) ( Figure 3e) is a widespread species that was discovered in lowland forest in French Guiana. ^89^ Surprisingly, the species has gone nearly a century without being recorded since Montagne proposed it. However, studies of ectomycorrhizal fungi in neotropical ecosystems have revealed that the species ranges from southern Brazil to northern Colombia. ^90^^,^^91^ Despite some minor morphological differences between the recorded specimens of *C. guyanensis* and the type specimen, they agree on the relevant character set. Its wide geographical distribution appears to reflect the wide variety of host plants. It can be found associated with monodominant forests of *Dicymbe* or *Aldina* spp. in Guyana; or multidiverse ectotrophic forests in spatial proximity to *Coccoloba*, *Guapira*, and *Neea* species in French Guiana, Colombia, Venezuela and Brazil (in the restinga). ^92^^,^^93^ Singer *et al.* ^48^ discovered *C. guyanensis* in the Brazilian Amazon in the 1980s, possibly associated with *Aldina* species as well as *Glycoxylon inophyllum* (Mart. ex Miq.) Ducke. Basidiomata of this species are solitary, abundant, visible only for a short period of time (for a month or so), ^91^ have an orange yellow to orange pileus, a hymenophore clearly laminated or regularly folded at all stages of growth and the presence of purplish tints in its predominantly orange pileus. ^90^

Considering all the *Clavulina* described for the restinga, *Clavulina junduensis* (MycoBank MB839651) ( Figure 3f) deserves attention. The species is characterized by the coralloid, branched, purplish grey basidiomata with brownish orange stipe; hyaline, subglobose to broadly ellipsoid basidiospores and abundant gloepleurous hyphae with refringent content and swelling bulbs. ^94^ Basidiomata of *C. junduensis* are frequently found in restinga fragments in southern Brazil but have been misidentified as *Clavulina cinerea* (Bull.) J. Schröt. for the past years. ^94^^–^^97^ However, considering that specimens with dark gray coloration do not group in a single clade in the phylogenies, studies suggest *C. cinerea* represents a species complex and more than one species with gray coloration is subsumed under this name. ^98^ Although we are still working on identifying the host, based on field observations, *C. junduensis* is possibly associated with *G. opposita*, which is the most common symbiont in restinga fragments in southern Brazil. ^33^ Another species associated with the restinga that also deserves a mention is *Clavulina incrustata* Wartchow (MycoBank MB561193) ( Figure 3g). The taxon was described by Wartchow ^99^ based on material collected in the Atlantic Forest from Pernambuco and it is the first species of *Clavulina* with incrusted hyphae. The presence of crystals in the specimens represents a character of taxonomic significance within the genus, and despite being microscopically identical, Tibpromma and colleagues ^100^ proposed *Clavulina paraincrustata* Meiras-Ottoni & Gibertoni to differentiate from specimens of *C. incrustata* with a less robust and pale basidiomata, with amphigenous hymenium. By the time of the publication, the DNA of the type specimen was tentatively extracted, but with no success. ^100^ However, the researchers recognized that the characters used to describe the new species have low taxonomic significance and, based on new phylogenetic analyzes, they proved to be the same species and synonymization of the names is expected [Angelina Meiras-Ottoni, personal communication]. According to the available data, *Clavulina* is an ancestrally tropical lineage, ^101^^,^^102^ and, although the genus can be found in a variety of ecosystems, it has been shown it is especially diverse in South America, where many new species have been recently described. ^11^^,^^103^^,^^104^

It is known that over the years and with the expansion of molecular phylogenetic analyzes of *Boletaceae*, the family has undergone several re-circumscriptions, with the rescue of some taxa and the segregation of others, ^105^^,^^106^ as in the case of the two newly proposed genera, *Brasilioporus* and *Nevesoporus.* ^74^ *Brasilioporus olivaceoflavidus* (MycoBank MB836726) ( Figure 3h), the type species of the genus, was collected in the coastal Atlantic Forest of the state of Espírito Santo, but it has also been recorded for the state of Santa Catarina, in restinga fragments. ^74^ This species has tiny basidiomata, a pileus with fibrils and olive-green scales on a yellowish background, and a blackish hymenophore when injured. It grows solitary and in small groups, or gregarious on sandy soil in the vicinity of ectomycorrhizal *Coccoloba, Guapira*, and *Pisonia* species. *Brasilioporus simoniarum* (MycoBank MB836727) ( Figure 3i) is a Brazilian species phylogenetically close to it. This species has been described as growing clustered in groups of three basidiomata on restinga sandy soil, in vicinity of *Guapira* spp. and it is known only from the type locality in the Brazilian Atlantic Forest on Florianópolis Island. ^74^ Different from *B. olivaceoflavidus*, *Brasilioporus simoniarum* have distinguished purplish black basidiomata, fibrillose to squamulose pileus, whitish hymenophore mottled orange-red and gradually turning black and subreticulate/sublacunose stipe. A beautiful, although discreet, species associated with restinga is *Nevesoporus nigrostipitatus* (MycoBank MB838704) ( Figure 3j), characterized by the small, basidiomata, with pinkish brown velvety pileus, pinkish tubes that are slightly depressed around the stipe and unchanging where bruised, and slender, dark gray to blackish stipe. ^74^ Although the species has only been found in the type locality, Espírito Santo, and Paraíba, it is expected to be found along the entire coast of the Atlantic Forest. *In situ*, *Nevesoporus nigrostipitatus* grows gregariously in small groups on sandy soil near species of *Coccoloba* and *Guapira.*

### Conservation issues

Despite their obvious relevance for understanding nature and ecosystem change, fungi have traditionally been neglected in biodiversity conservation. However, a number of initiatives and studies have raised general attention toward the status of fungal populations across the world, increasing awareness and spurring protection actions dedicated to fungi. ^107^^,^^108^ Restinga makes no exception, and together with the observation and description of its fungal diversity, it comes the assessment of the conservation status of several ectomycorrhizal taxa. *A. viscidolutea*, for example, has been reported from a handful of sites, although it is likely to be more widespread. A population decline of between 30% and 50% within the last three generations (50 years) has been suspected, based on the severe habitat decline in the area, justifying its conservation assessment as ‘Vulnerable’ following the IUCN criteria. ^109^ Another species determined as Vulnerable is *A. festivus.* ^110^^,^^111^ Known from the coastal Atlantic Forest of Brazil (Pernambuco, Paraná and Santa Catarina states), *A. festivus* occurs solitary to scattered in white sandy soil under trees in restinga. “There is concern over a decline of the habitat considering the restinga areas, as they are small highly fragmented patches open to recreational activities and tourism and there are no strict laws that restrict the use of these areas. Also, the areas in southern and north-eastern Brazil have been impacted by urban growth, threatening the last remnants of Atlantic Coast restingas. Invasion by non-native pine ( *Pinus elliottii*) is another threat,” reads the original description of the threats menacing this species. ^111^ Devising measures to efficiently protect threatened fungal restinga species is not a trivial matter. Generally speaking, protection of habitats where endangered macrofungi are found is pivotal for the conservation of these key microorganisms. To this aim, curbing the spread of invasive non-native species and avoiding excessive human exploitation of coastal areas are key conservation actions, especially when coupled to sound data on the distribution and population size of the macrofungi object of protection.

## Conclusions

Restinga mycorrhizal biology and ecology is under the spotlight, but clearly, we are just scratching the surface. Identity of host plants, host-specificity of associated mycobionts and patterns of shared mycorrhizal networks among host plants, the role played by dual mycorrhizal symbiosis, are only a few of the many aspects that demand further investigation. ^112^ Besides enhancing our basic understanding of restinga as an ecosystem, casting light on these issues would also have practical consequences. Identifying symbionts and their effects on ecosystems, for example, will enable the development of conservation and restoration strategies for the restinga. Hopefully, this and other works will increase the awareness of researchers, providing us in the near future with fresh data coming from both fungal and botanical forays, aimed at describing the diversity of ECM fungi and associated plant ecology in restinga. Also, well-planned molecular studies examining mycorrhizal specificity at the root tip scale are bound to disclose many details of the structure and dynamics of restinga ectomycorrhizal communities.

## Author Contributions

All authors made substantial contributions to the conceptualization and design of the work, drafting the work and critically reviewing it for important intellectual content, as well as all authors approved the final version for publication. ANMF, MAN and ACR supervised the planning and execution of the research, including external guidance from the core team. ANMF annotated and curated the data for its initial use and later reuse, checking the overall reproducibility of the results. ANMF, ML and OC conducted phylogenetic analysis and morphological observations. MAN, ACR, ML and OC provided study materials and all other resources used as analysis tools. ACR, OC and ML secured financial support for the project that gave rise to this publication. All authors directed and coordinated the planning and execution of the research activity.

## Data Availability

GenBank: Eukaryotic Nuclear rDNA/ITS/Restinga ectomycorrhizas/Tomentella sp. isolate M30R173. Accession number OP819286; https://identifiers.org/ncbi/insdc:OP819286. ^145^ GenBank: Eukaryotic Nuclear rDNA/ITS/Restinga ectomycorrhizas/Tomentella sp. isolate M34R198. Accession number OP819287; https://identifiers.org/ncbi/insdc:OP819287. ^146^ GenBank: Eukaryotic Nuclear rDNA/ITS/Restinga ectomycorrhizas/Tomentella sp. isolate M36AR200a. Accession number OP819288; https://identifiers.org/ncbi/insdc:OP819288. ^147^ GenBank: Eukaryotic Nuclear rDNA/ITS/Restinga ectomycorrhizas/Tomentella sp. isolate M36AR200b. Accession number OP819289; https://identifiers.org/ncbi/insdc:OP819289. ^148^ GenBank: Eukaryotic Nuclear rDNA/ITS/Restinga ectomycorrhizas/Austroboletus festivus isolate M41CR210. Accession number OP819290; https://identifiers.org/ncbi/insdc:OP819290. ^149^ GenBank: Eukaryotic Nuclear rDNA/ITS/Restinga ectomycorrhizas/Inocybe sp. isolate M51AR230. Accession number OP819291; https://identifiers.org/ncbi/insdc:OP819291. ^150^ GenBank: Eukaryotic Nuclear rDNA/ITS/Restinga ectomycorrhizas/Thelephoraceae isolate M53AR235. Accession number OP819292; https://identifiers.org/ncbi/insdc:OP819292. ^151^ GenBank: Eukaryotic Nuclear rDNA/ITS/Restinga ectomycorrhizas/Tomentella sp. isolate M57BR248. Accession number OP819293; https://identifiers.org/ncbi/insdc:OP819293. ^152^ GenBank: Eukaryotic Nuclear rDNA/ITS/Restinga ectomycorrhizas/Tomentella sp. isolate M68AR291. Accession number OP819294; https://identifiers.org/ncbi/insdc:OP819294. ^153^ Figshare: Collections of ectomycorrhizal fungi from restinga fragments on the Brazilian coast. https://doi.org/10.6084/m9.figshare.22196836. ^23^ This project contains the following underlying data:
‐ Furtado ANM et al - @F1000 Res_Supplementary Table 1.xlsx. Data are available under the terms of the Creative Commons Zero “No rights reserved” data waiver (CC0 1.0 Public domain dedication).
