# Supplementary material for: Restinga ectomycorrhizae: a work in progress
Source: F1000Res. 2023 Mar 22;12:317. [Version 1] doi: 10.12688/f1000research.131558.1 (PMC10230178; doi:10.12688/f1000research.131558.1)
Supplement: Peer review history

# Peer review: Reviewer response for version 1

**DOI:** 10.5256/f1000research.144410.r167497

**Published:** 2023-05-30

**Authors:** Roberto Flores Arzù

**Affiliations:** 1 Unidad de Biodiversidad, Tecnología y Aprovechamiento de Hongos—UBIOTAH—, Departamento de Microbiología, Facultad de Ciencias Químicas y Farmacia, Universidad de San Carlos de Guatemala, Guatemala City, Guatemala Department, Guatemala

This article proves the importance of the Restinga Forest of Brazil, a not well-known ecosystem, whose mycobiota astonish with the presence of ectomycorrhizal fungi, with common genera present in Northern Hemisphere and temperate regions as Tomentella and Thelephora, but also with new records of basidiomicetes in Amanita, Austroboletus, Clavulina, Inocybe and Russula. The study verifies the identity of the fungal symbionts not only by the fruit bodies but also by different ectomycorrhizhae in restinga soil and by molecular analyses using the ITS region, adequate but not exhaustive to separate fungal genera and species.

 The results remind the mycobiota found in the Guyana Shield, a region with similar tree genera but with the description of a new mycorrhiza type: the guapiroid, formed by Guapira and some fungal genera. A good discussion is introduced about coexistence and the role of AMF and ECM fungi in the Restinga Forest, as well as an accurate review of the distribution of the South American ectomycorrhizal species found.

 The results are very interesting for the fungal diversity found. Authors humbly say that they are “just scratching the surface” of the Brazilian Restinga Forest and it is quite possible that diversity will be larger.

 1. The work is clear, accurately presented and cites current literature with novel results for ECM fungi in South America.

 2. Appropriate study design, considering the references and the sequences used in the analyses and conclusions.

 3. Methodology sufficiently detailed and analysis provided can be replicated by others. There were no new techniques applied but a well-documented study to support the results.

 4. The conclusions are quite supported by the results due to the deep bibliographic analysis about fungi and symbiont plants in Tropical South America.

 5. Some minor changes could improve the paper as linking better the phrases in the first paragraph of Introduction.

 6. The Figure 2 could be omitted if published in references 33 or 50.

 7. Authors point correctly that genus *Boletus *has not been recorded in Brazil except from exotic plantations.

 8. Maybe they could say something about the two *Phlebopus *species cited in the database, considering that both species have an African origin?

 9. There is a good analysis for *Boletellus *in Brazil as well as for *Amanita *and *Clavulina.*

Is the work clearly and accurately presented and does it cite the current literature?

Yes

If applicable, is the statistical analysis and its interpretation appropriate?

Not applicable

Are all the source data underlying the results available to ensure full reproducibility?

Yes

Is the study design appropriate and is the work technically sound?

Yes

Are the conclusions drawn adequately supported by the results?

Yes

Are sufficient details of methods and analysis provided to allow replication by others?

Yes

Reviewer Expertise:

Ectomycorrhizal mushrooms of the Neotropics, especially in Subtropical lands. Ethnomycology. Ectomycorrhizae.

I confirm that I have read this submission and believe that I have an appropriate level of expertise to confirm that it is of an acceptable scientific standard.

# Peer review: Reviewer response for version 1

**DOI:** 10.5256/f1000research.144410.r168665

**Published:** 2023-04-24

**Authors:** Julieta Álvarez Manjarrez

**Affiliations:** 1 Universidad Nacional Autonoma de Mexico, Mexico City, Mexico City, Mexico

The paper is a very important piece of information about the fungal diversity of a natural ecosystem in Brazil. The sampling reported 726 records from ectomycorrhizal taxa with the identification of their hosts. The paper is very well written and I just added few comments to improve it:

- *“In Brazil, ectomycorrhizal fungi often have a fragmented distribution due to the lack of information about them and because they do not always have the same distribution as the host plants”*
  It is necessary to rephrase the two ideas of this sentences: the estimation of ECM fungi distribution is fragmented according to the current information; and how is it possible that the distribution have not the same distribution of host plants, I think you mean there is still missing information about all the ECM hosts in Brazil. Also, the citation you used to argue this sentence came from other countries but you are talking particularly from Brazil, could you add some accurate reference for Brazil ECM fungi and their hosts?
- *"This can result in high endemism at the species level due to the specific habitats they occupy."*
  For me is not clear how the lack of information, that is the main topic of the previous sentence, would produce high endemism. Please be more clear about this hypothesis.
- *"In 2016, Roy and coworkers reported approximately 180 species of ectomycorrhizal fungi in Brazilian native forests. ^15^ In fact, the majority of the published studies were conducted in introduced Pinus and Eucalyptus plantations."*
  The writing is very confusing, first you mentioned the number of species in native forests, but then you jumped to talk about plantations. I recommend to remove the plantation sentence from this paragraph and try to write a better connection between all the ideas form this paragraph.
- In Restinga definition, please provide information about the soil types.
- For Figure 1 it would be more useful to be closer to the areas that have this vegetation instead of presenting all Latin American map, and indicate with a signal the places you studied.
- Add the GenBank number accession of the ITS region from the plant.
- *“The roots were washed and carefully selected under a stereomicroscope.” - *Which characteristics you observed to select the root tips?
- How did you extract DNA from the ECM tips?
- In Table 1, would be ideal to know the location of every fungal species collections. Also it is unclear how you add more than one host per ECM fungal species, 1) how did you obtain more than 1 host? Then, when you have a list of hosts, use the asterisk for every single host name to refers to potential host. For example: Euphorbiaceae, Fabaceae, Mimosaceae*, does that mean that just Mimosaceae is not confirmed or the whole plant list?
- Add the GenBank number accession of your data in the Table 1. And add a column saying if the sequences were obtained from fruitbodies or ECM root tips.
- I found some plant names are taxonomically incorrect, for example Mimosaceae or Caesalpiniaceae. Confirm that all the plant species/family names you are using are correct. You can use theplantlist.org or tropicos.org. I encourage that if there is a list of plants from the restinga, provide it in a Support information or at least a link where we can read the possible host names in each plant family you mentioned.
- What is the percentage of similarity between your specimens of *A. crebresulcata* and GenBank? Could they be a different species?
- “Considering all the *Clavulina* described for the restinga” add citations
- Add scale bar to each photo in Figures 2 and 3.
- Provide an anatomical photo from the EM of *Guapira opposita* and cited after *“The ectomycorrhizal morphotypes we observed on G. opposita do not present the intraradical hyphae arrangement observed in other Nyctaginaceae. However, the absence of Hartig net recorded in our study is another indication of the plasticity and peculiarity of mycorrhizal biology of this host plant family.”*
- You mentioned the AMF in restinga had been studied; besides *G. opposita*, do you know if your EM hosts also have AM in their roots?

Is the work clearly and accurately presented and does it cite the current literature?

Yes

If applicable, is the statistical analysis and its interpretation appropriate?

Not applicable

Are all the source data underlying the results available to ensure full reproducibility?

Yes

Is the study design appropriate and is the work technically sound?

Yes

Are the conclusions drawn adequately supported by the results?

Yes

Are sufficient details of methods and analysis provided to allow replication by others?

Partly

Reviewer Expertise:

Tropical ecology and fungal systematics, especially ectomycorrhizal fungi

I confirm that I have read this submission and believe that I have an appropriate level of expertise to confirm that it is of an acceptable scientific standard.
